# Incidence and risk factors of postoperative complications following periodontal flap surgery: a retrospective study

**DOI:** 10.3389/fdmed.2025.1668844

**Published:** 2025-09-24

**Authors:** Hongjiang Gu, Wei Liu, Xiaoyu Xu, Yinjie Shen

**Affiliations:** Department of Stomatology, Taicang Hospital of Traditional Medicine, Taicang, Jiangsu, China

**Keywords:** periodontal flap surgery, complication, clinical attachment loss, risk factors, logistic regression

## Abstract

**Background:**

Periodontal flap surgery is a common procedure for treating advanced periodontal disease, but postoperative complications such as pain, infection, and delayed healing are frequent. This study aims to investigate the incidence of postoperative complications and identify key risk factors, contributing to more personalized surgical management.

**Methods:**

A retrospective analysis was conducted on 121 patients who underwent periodontal flap surgery. Patient demographics, clinical data (e.g., age, probing depth, clinical attachment loss), and surgical variables (e.g., incision type, surgery duration) were collected. Logistic regression analysis was performed to identify significant predictors of postoperative complications. The complications analyzed included dentin hypersensitivity, excessive pain, infection, and gingival bleeding.

**Results:**

Postoperative complications occurred in 44.63% of patients. Age was a significant predictor, with each additional year increasing the risk of complications by 12% (*p* < 0.001). Clinical attachment loss was strongly associated with complications (*p* = 0.009). Semilunar incisions, typically used in localized mild cases, were associated with a lower incidence of complications compared to trapezoidal incisions (*p* < 0.001). Smoking also significantly increased the risk of complications in both the univariate and multivariate analyses (*p* < 0.05). In the univariate analysis, both preoperative antibiotics and preoperative analgesics were associated with a reduced risk of complications (*p* < 0.05), but these effects were not significant in the multivariate analysis (*p* > 0.05).

**Conclusion:**

This study highlights age, clinical attachment loss, smoking, and surgical incision type as significant predictors of postoperative complications following periodontal flap surgery. The findings underscore the need for personalized surgical approaches, particularly in older patients and those with severe periodontal disease.

## Introduction

Periodontal disease is a prevalent chronic inflammatory condition that affects the supporting structures of teeth, including the gingiva, alveolar bone, and periodontal ligament (1). It is one of the leading causes of tooth loss in adults, with moderate to severe periodontitis affecting a significant proportion of the global population (2). Treatment of advanced periodontal disease often requires surgical intervention, with periodontal flap surgery being one of the most common procedures aimed at controlling infection, reducing pocket depths, and regenerating lost periodontal tissues (3).

Periodontal flap surgery involves making incisions in the gingiva to gain access to the root surfaces and underlying alveolar bone for thorough debridement and tissue regeneration (4). Although this surgical technique is effective in managing periodontal disease, it is associated with a risk of postoperative complications such as dentin hypersensitivity, excessive pain, infection, gingival bleeding, wound dehiscence, and delayed healing (5, 6). These complications can adversely affect patient outcomes, prolong recovery times, and, in some cases, necessitate additional interventions (5).

The occurrence of postoperative complications following periodontal flap surgery is influenced by a range of patient-related and clinical factors. Previous studies have identified factors such as patient age, smoking status, diabetes, and the severity of periodontal disease as potential risk factors for adverse outcomes (7–9). However, there is still a need to further explore the impact of various demographic, clinical, and surgical variables on complication rates, particularly in different patient populations and clinical settings.

The aim of this retrospective study is to investigate the incidence and risk factors associated with postoperative complications following periodontal flap surgery. Specifically, we aim to evaluate the influence of demographic characteristics (e.g., age, gender, BMI), clinical factors (e.g., probing depth, clinical attachment loss), and surgical variables (e.g., incision type, surgery duration) on the likelihood of complications. By identifying significant predictors of complications, we seek to provide valuable insights for clinicians to optimize patient outcomes and reduce the incidence of adverse events in periodontal surgery.

## Methods

### Study design and participants

This retrospective study was conducted at Taicang Traditional Chinese Medicine Hospital and included 121 patients who underwent periodontal flap surgery between January 2022 and December 2023. Patients were selected based on the following inclusion criteria: 1. diagnosed with moderate to severe periodontitis and treated with periodontal flap surgery; 2. aged 18 years or older; and 3. had complete clinical data and oral examination records available. Patients were excluded if they: 1. had coexisting oral diseases such as severe dental caries or oral cancer; 2. suffered from oral trauma or injuries; 3. had severe immune system disorders or infectious diseases; 4. had a history of periodontal surgery; or 5. had incomplete or missing clinical data; or 6. had a history of chronic systemic conditions (e.g., rheumatoid arthritis, chronic musculoskeletal disorders) requiring long-term analgesic use, to minimize potential confounding effects. The study protocol was approved by the Institutional Review Board of our hospital (Ethical Approval Number: TAIZHONG-2023035).

### Surgical indication criteria

The decision to perform periodontal flap surgery was based on specific site-based criteria rather than whole-mouth averages. Patients selected for surgery typically had localized deep periodontal pockets (PD ≥ 5 mm) that did not respond to non-surgical therapy. Additionally, clinical attachment loss (CAL ≥ 3 mm) and radiographic evidence of bone loss were also considered for surgery. These criteria are in line with established clinical guidelines for periodontal surgery, ensuring that the procedures were only performed on patients with clinically significant periodontal disease.

### Case selection

To provide a detailed example of the procedure and its outcomes, we highlight one representative case from the study. This case involves a 43-year-old female patient who had been experiencing left lower posterior tooth mobility for approximately one year. The patient presented with a full mouth plaque index of I°–II°, gingival bleeding (BOP+), and soft plaque (+), along with minimal gingival edema. Upon clinical examination, the right lower 34th tooth showed a periodontal pocket depth (PD) of 5–7 mm, with tooth mobility of degree I in tooth 35 and degree II in tooth 34. Radiographic evidence revealed significant periodontal damage, and conventional non-surgical treatment was ineffective. As a result, the patient was selected for periodontal flap surgery to remove diseased tissue and restore periodontal health. This case will serve as a detailed example throughout the study to demonstrate the clinical process and surgical outcomes.

### Surgical procedure

Conventional periodontal flap surgery was used in the present study. The procedure began with a thorough preoperative assessment, including radiographic imaging, to evaluate the extent of periodontal damage. Prior to the surgery, all patients underwent non-surgical treatment, including scaling and root planing (SRP) to remove plaque and calculus, and local antimicrobial therapy to control inflammation. Local anesthesia was administered to ensure patient comfort during the surgery.

Small incisions were made in the gingiva to expose the affected root surfaces and alveolar bone. Specialized instruments, such as ultrasonic scalers or lasers, were used to meticulously remove plaque, calculus, and diseased tissue. In cases requiring regeneration, bone grafts or guided tissue regeneration membranes were applied to promote tissue healing. For the gingival margins, a horizontal mattress suture combined with an interrupted suture was applied to secure the tissue, particularly at the interdental papilla, ensuring good coverage and minimizing tension.

Postoperative care generally included antibiotics, pain management, and specific oral hygiene instructions, though antibiotic use was determined based on individual patient needs, surgical complexity, and postoperative complication risk. Specifically, patients were prescribed amoxicillin 500 mg three times daily for 5 days, flurbiprofen 100 mg twice daily for 3 days, and instructed to use 0.2% chlorhexidine gluconate mouth rinse twice daily for 7 days. Patients were advised to begin the mouth rinse 24 h after surgery and to maintain a soft diet for the first 24 h. Normal oral hygiene was resumed the following day. All patients underwent standardized periodontal flap surgery primarily aimed at debridement. While regenerative adjuncts such as bone grafts or membranes were used in selected cases, the procedures were not classified into distinct surgical types for analysis, to maintain focus on a unified surgical context.

The surgery involved two types of incisions: a semilunar incision and a trapezoidal incision. The semilunar incision was primarily used for mild cases with localized lesions, typically involving one or two adjacent teeth. The incision followed a curved line along the gingival margin and was confined to the mucosal layer, constituting a shallow mucosal-level flap design. The trapezoidal incision, on the other hand, was used for more extensive cases, involving multiple teeth, with the incision extending deeper into the attached gingiva. Both types of incisions were performed with the goal of achieving optimal access for debridement while minimizing tissue trauma. The location and extent of the incisions were determined based on the clinical need for each patient, considering the affected teeth and the severity of the periodontal disease.

### Data collection

The following demographic and clinical variables were extracted from patient records: age, gender, BMI, smoking status (categorized as current smoker or non-smoker), alcohol consumption (current or none), diabetes status (yes or no), hypertension (yes or no), dyslipidemia (yes or no), coronary artery disease (yes or no), preoperative use of antibiotics (yes or no), preoperative use of analgesics (yes or no), gingival bleeding index, probing depth, clinical attachment loss, surgical incision type (semilunar incision or traditional trapezoidal incision), and surgery duration, where the start and end times of each procedure were routinely documented by the surgical team. Alcohol consumption was defined as “current” if the patient drank daily in the 6 months prior to the study, and “none” if they did not; diabetes was “yes” if the patient had been diagnosed with diabetes, and “no” if not; hypertension was “yes” if the patient had a history of high blood pressure or a blood pressure reading ≥140/90 mmHg; dyslipidemia was “yes” if lipid levels were abnormal; coronary artery disease was “yes” if diagnosed through medical history or imaging.

### Postoperative complications

Postoperative complications were recorded and included dentin hypersensitivity, excessive pain, fistula formation, infection, gingival bleeding, redness and swelling, wound dehiscence, gingival recession, bone loss, and delayed healing. These complications were assessed during scheduled follow-up visits on postoperative days 1, 3, and 7, and during the 1-month follow-up appointment.

Pain levels were evaluated using a visual analog scale (VAS) scored from 0 (no pain) to 10 (worst pain), and were self-reported by patients. Dentin hypersensitivity was assessed with a cold stimulation test, rated from 0 (no sensitivity) to 3 (severe sensitivity). Fistula formation and infection were identified through clinical examination, with infection confirmed by the presence of pus, redness, swelling, or increased temperature at the surgical site.

Gingival bleeding was assessed using the Gingival Bleeding Index (GBI), ranging from 0 (no bleeding) to 3 (significant bleeding). Redness and swelling were visually inspected and graded from 0 (none) to 3 (severe). Wound dehiscence was evaluated based on clinical observation of the wound's healing status. Gingival recession was measured using probing depth changes. Bone loss was assessed through radiographic analysis, and delayed healing was identified by clinical observation of prolonged wound healing or complications. All findings were documented by trained periodontists using standardized criteria and recorded in the patients' electronic medical records for further analysis.

### Statistical analysis

To identify potential risk factors for postoperative complications, a logistic regression analysis was conducted. The variables included in the analysis were age, gender, BMI, smoking status, alcohol consumption, diabetes, hypertension, dyslipidemia, coronary artery disease, preoperative use of antibiotics, preoperative use of analgesics, gingival bleeding index, probing depth, clinical attachment loss, surgical incision type, and surgery duration. The results were expressed as odds ratios with corresponding confidence intervals and *p*-values, determining the significance of each variable as a risk factor for postoperative complications. Additionally, model fit was evaluated using the Hosmer–Lemeshow test, and pseudo-*R*^2^ values (Nagelkerke *R*^2^) were calculated to assess explanatory power. Variance inflation factor (VIF) and tolerance diagnostics were performed to detect multicollinearity.

## Results

### Patient characteristics

A total of 121 patients who underwent periodontal flap surgery were included in this study. The mean age of the patients was 58.59 ± 8.71 years, with a nearly equal distribution of males (49.59%) and females (50.41%). Most patients (97.52%) had a BMI of less than 30, while only 2.48% had a BMI of 30 or higher. Current smokers accounted for 69.42% of the sample, and 18.18% of patients reported alcohol use. Among the patients, 18.18% had diabetes, 27.27% had hypertension, and 24.79% had dyslipidemia. Additionally, 14.05% of the patients had coronary artery disease. Preoperative antibiotics were used in 50.41% of cases, and preoperative analgesics were used in 56.20%. Clinical data showed an average probing depth of 3.33 ± 0.76 mm and a clinical attachment loss of 2.72 ± 0.69 mm. The surgical incision type was semilunar in 52.07% of the cases, while the remaining 47.93% underwent a trapezoidal incision. The average surgery duration was 45.67 ± 9.77 min (Table 1).

**Table 1:** Patient characteristics.

Characteristic	*n* = 121
Age (years)	58.59 ± 8.71
Gender (M/F)	60 (49.59%)/61 (50.41%)
BMI	
<30	118 (97.52%)
≥30	3 (2.48%)
Current smokers	
Yes	84 (69.42%)
No	37 (30.58%)
Current alcohol use	
Yes	22 (18.18%)
No	99 (81.82%)
Diabetes	
Yes	22 (18.18%)
No	99 (81.82%)
Hypertension	
Yes	33 (27.27%)
No	88 (72.73%)
Dyslipidemia	
Yes	30 (24.79%)
No	91 (75.21%)
Coronary artery disease	
Yes	17 (14.05%)
No	104 (85.95%)
Preoperative antibiotics	
Yes	61 (50.41%)
No	60 (49.59%)
Preoperative analgesics	
Yes	68 (56.20%)
No	53 (43.80%)
Clinical Attachment Loss (mm)	2.72 ± 0.69
Gingival bleeding index	1.89 ± 0.55
Probing depth (mm)	3.33 ± 0.76
Surgical incision type	
Semilunar	63 (52.07%)
Trapezoidal	58 (47.93%)
Surgery duration (min)	45.67 ± 9.77

### Postoperative complications

Postoperative complications occurred in 44.63% (54/121) of patients (Table 2). The most common complication was dentin hypersensitivity, observed in 10.74% of patients. Excessive pain was reported in 7.44% of cases, while fistula formation, infection, gingival bleeding, and redness and swelling occurred in smaller proportions, ranging from 0.83% to 9.92%. Wound dehiscence, gingival recession, bone loss, and delayed healing were less frequent complications, each affecting less than 5% of the patients.

**Table 2:** Postoperative complications.

Complication	*n* = 54
Dentin hypersensitivity	13 (10.74%)
Excessive pain	9 (7.44%)
Fistula formation	1 (0.83%)
Infection	4 (3.31%)
Gingival bleeding	3 (2.48%)
Redness and swelling	12 (9.92%)
Wound dehiscence	2 (1.65%)
Gingival recession	3 (2.48%)
Bone loss	2 (1.65%)
Delayed healing	5 (4.13%)

### Logistic regression analysis

The univariate logistic regression analysis identified several variables significantly associated with the occurrence of postoperative complications following periodontal flap surgery. As shown in Table 3, age emerged as a significant risk factor, with each additional year of age increasing the risk of complications by 12.8% (OR: 1.128, 95% CI: 1.067–1.192, *p* < 0.001). This finding remained significant in the multivariate analysis (OR: 1.126, 95% CI: 1.060–1.205, *p* < 0.001), indicating that age is an independent risk factor. Clinical attachment loss also showed a strong association with postoperative complications. In the univariate analysis, it was found to significantly increase the risk of complications (OR: 3.264, 95% CI: 1.759–6.055, *p* < 0.001), and this remained significant in the multivariate analysis (OR: 2.761, 95% CI: 1.319–6.262, *p* = 0.010).

**Table 3:** Logistic regression analysis.

Variables	Univariate Analysis OR (95% CI)	*P*	Multivariate Analysis OR (95% CI)	*P*
Age	1.128 (1.067–1.192)	0.000	1.126 (1.060–1.205)	0.000
Sex	1.347 (0.656–2.764)	0.417		
BMI	1.060 (0.937–1.198)	0.354		
Gingival bleeding index	1.969 (0.995–3.896)	0.052		
Probing depth	1.734 (1.045–2.877)	0.033	0.880 (0.437–1.747)	0.715
Clinical Attachment Loss	3.264 (1.759–6.055)	0.000	2.761 (1.319–6.262)	0.010
Surgical incision type	5.581 (2.548–12.227)	0.000	7.375 (2.725–22.393)	0.000
Surgery duration	1.016 (0.979–1.055)	0.401		
Current smokers	3.590 (1.512–8.524)	0.004	5.070 (1.635–17.844)	0.007
Current alcohol use	1.302 (0.516–3.286)	0.576		
Diabetes	0.831 (0.325–2.121)	0.698		
Hypertension	1.238 (0.555–2.763)	0.602		
Dyslipidemia	1.116 (0.487–2.554)	0.796		
Coronary artery disease	1.121 (0.401–3.133)	0.828		
Preoperative antibiotics	0.477 (0.227–0.985)	0.048	0.635 (0.230–1.733)	0.374
Preoperative analgesics	0.447 (0.212–0.924)	0.031	0.501 (0.180–1.347)	0.174

The type of surgical incision also played a critical role in determining the likelihood of complications. Patients who underwent semilunar incisions had a significantly lower risk of complications compared to those with trapezoidal incisions. This was evident in both the univariate analysis (OR: 5.581, 95% CI: 2.548–12.227, *p* < 0.001) and the multivariate analysis (OR: 7.375, 95% CI: 2.725–22.393, *p* < 0.001). Smoking was found to have a risk effect against postoperative complications, with smokers showing a high risk in both the univariate (OR: 3.590, 95% CI: 1.512–8.524, *p* = 0.004) and multivariate analyses (OR: 5.070, 95% CI: 1.635–17.844, *p* = 0.007).

In the univariate analysis, both preoperative antibiotics (OR: 0.477, 95% CI: 0.227–0.985, *p* = 0.048) and preoperative analgesics (OR: 0.447, 95% CI: 0.212–0.924, *p* = 0.031) were associated with a reduced risk of postoperative complications. However, these associations were not significant in the multivariate analysis, with antibiotics (OR: 0.635, 95% CI: 0.230–1.733, *p* = 0.374) and analgesics (OR: 0.501, 95% CI: 0.180–1.347, *p* = 0.174) showing no independent protective effects. Other factors, such as gender, BMI, gingival bleeding index, probing depth, and comorbidities like diabetes, hypertension, and coronary artery disease, did not show significant associations with postoperative complications in either the univariate or multivariate analyses (*P* > 0.05). Additionally, the Hosmer–Lemeshow test indicated good model fit (*χ*^2^ = 6.87, *p* = 0.551). The Nagelkerke *R*^2^ value was 0.545, suggesting a moderate explanatory power. All covariates included in the multivariate analysis had VIF values <2, indicating no evidence of significant multicollinearity.

### Periodontal flap surgery treatment process and postoperative outcomes

**Figure 1:**
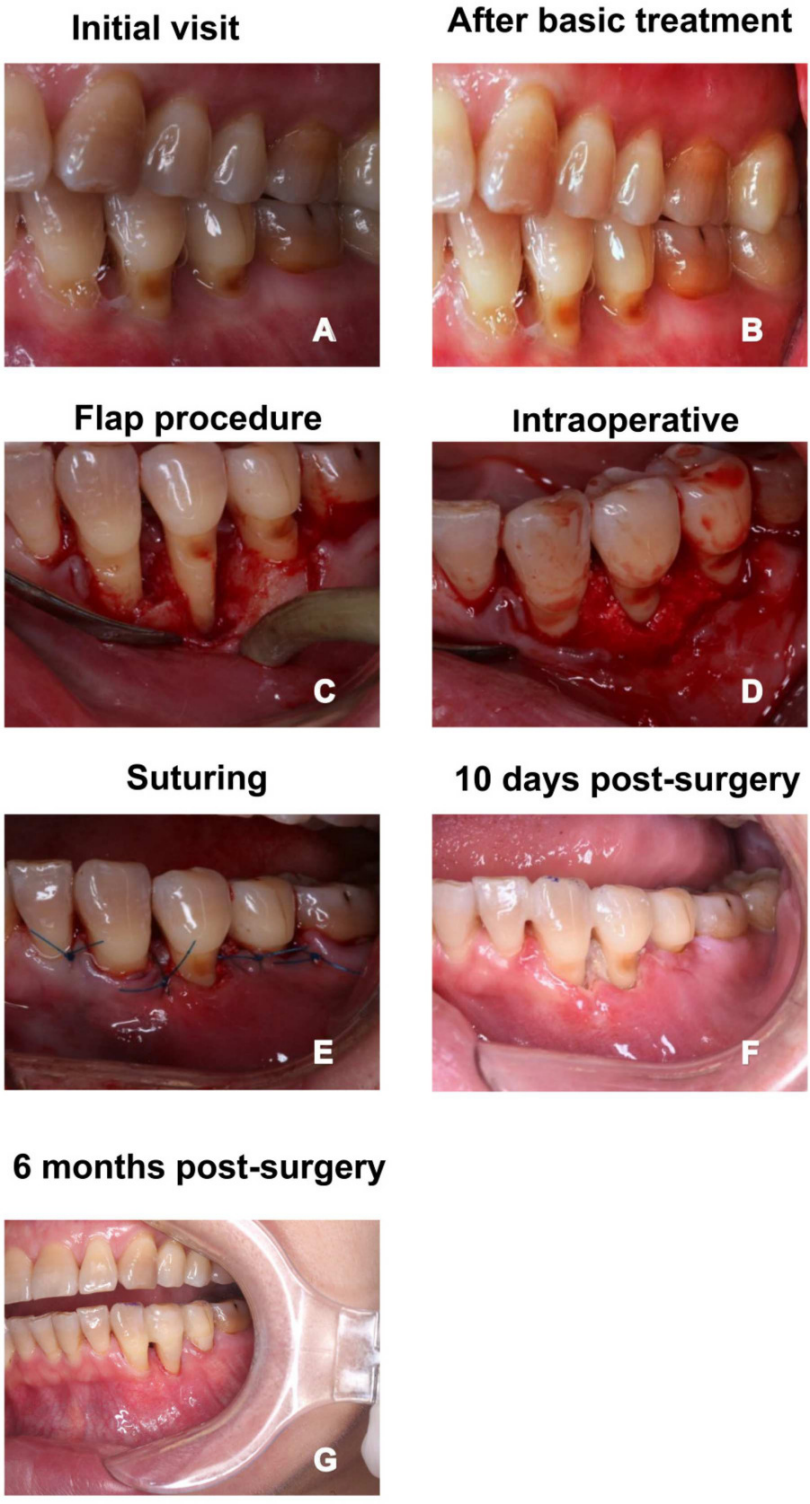
One case of conventional periodontal flap surgery. **(A)** Clinical appearance at the initial visit; **(B)** gingival condition after basic treatment; **(C)** periodontal flap surgery procedure, exposing the root surfaces and alveolar bone; **(D)** intraoperative view showing the treated periodontal lesion area; **(E)** suturing phase after surgery, with gingiva repositioned for optimal healing; **(F)** 10 days post-surgery, showing the healing status of the surgical site; **(G)** 6 months post-surgery, with stable gingival health and recovery.

**Figure 2:**
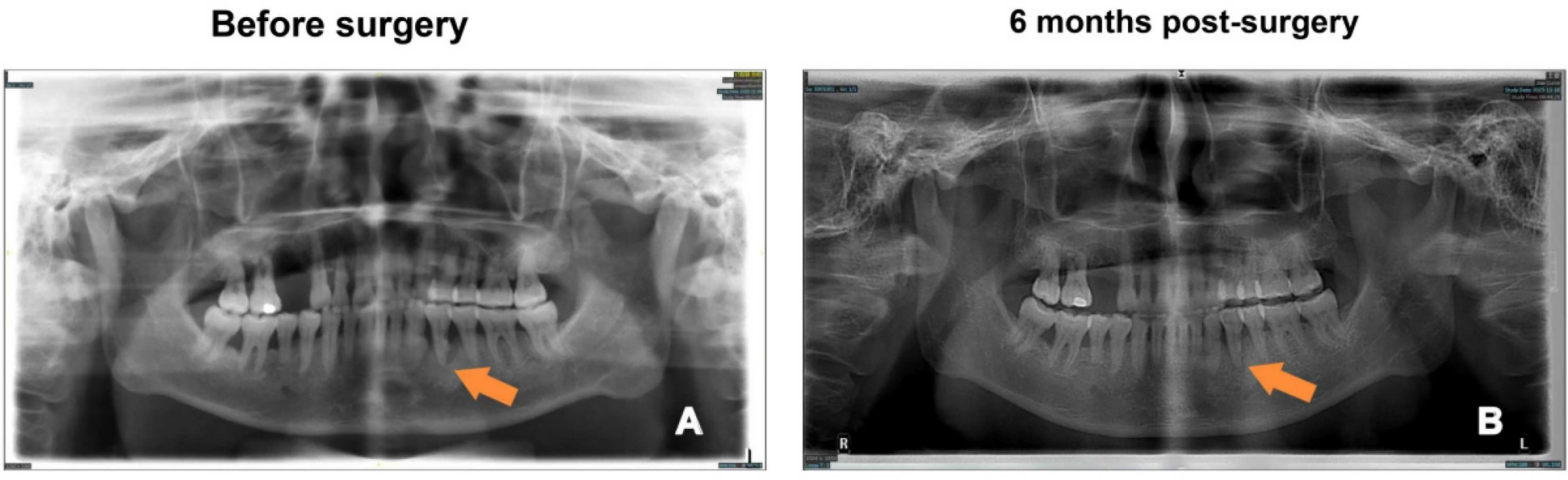
Radiographic comparison of periodontal health before and after surgery. **(A)** Preoperative radiograph showing the periodontal condition before surgery (arrow indicates the area of concern); **(B)** Radiograph taken 6 months post-surgery, showing improvement in periodontal health (arrow indicates the healed area).

The patient showed significant improvement in clinical condition following periodontal flap surgery. From the initial visit to 6 months post-surgery, the patient's gingival condition exhibited notable changes. At the initial visit (Figure 1A), the patient had considerable gingival edema, deep periodontal pockets, gingival bleeding (BOP+), and plaque accumulation. After basic treatment (Figure 1B), there was a reduction in gingival edema, although some localized disease areas persisted. During the surgery (Figures 1C,D), the affected root surfaces and alveolar bone were successfully exposed, and plaque, calculus, and diseased tissue were meticulously removed. The gingiva was then repositioned and sutured (Figure 1E) to ensure optimal healing, with proper fixation to prevent tension. At 10 days post-surgery (Figure 1F), initial signs of healing were evident, with reduced inflammation and a more normal gingival contour. By 6 months post-surgery (Figure 1G), the gingiva had fully recovered, and alveolar bone loss was effectively controlled, confirming the success of the surgical procedure in restoring periodontal health. In addition to the clinical observations, radiographic images (Figure 2) further demonstrate the improvement in periodontal health. Preoperative radiographs (Figure 2A) revealed significant bone loss and periodontal pockets. However, 6 months post-surgery (Figure 2B), the radiographic images clearly indicate substantial healing, with reduced periodontal pockets and improved bone structure, supporting the positive outcomes of the surgical intervention.

## Discussion

This retrospective study identified key risk factors for postoperative complications following periodontal flap surgery, with age, clinical attachment loss, smoking, and surgical incision type emerging as significant predictors. These findings enhance our understanding of the variables influencing surgical outcomes and offer important insights for optimizing patient care.

The association between age and complications aligns with previous studies, which have consistently shown that older patients face a higher risk of adverse outcomes (10, 11). With advancing age, tissue regenerative capacity decreases, and comorbidities such as diabetes and hypertension become more prevalent, impairing wound healing and exacerbating inflammation (12, 13). The present study reaffirms that older patients are more susceptible to complications, emphasizing the need for tailored surgical approaches in this population.

Clinical attachment loss was another significant factor, indicating that the severity of periodontal disease directly influences surgical outcomes. Greater attachment loss correlates with more extensive tissue destruction, which increases the likelihood of postoperative complications such as infection and wound dehiscence (14). Suvan et al. (15) similarly have reported that higher clinical attachment loss is linked to poorer healing outcomes in periodontal surgery, supporting the need for more cautious postoperative management in patients with severe disease.

Smoking was identified as a significant risk factor for postoperative complications, which aligns with the well-documented harmful effects of smoking on wound healing and tissue regeneration (16, 17). In our study, 69.42% of patients were current smokers, which is not uncommon in our regional clinical population, particularly among middle-aged and older adults with a history of periodontitis. This high proportion reflects the local epidemiological pattern of tobacco use, suggesting that the findings are generalizable within similar patient populations. Smoking is known to decrease oxygen delivery to tissues, impair immune function, and delay healing processes, thereby increasing the likelihood of infections, wound dehiscence, and other complications (18, 19).

The surgical incision type also plays a critical role. Semilunar incisions were found to reduce the risk of complications compared to trapezoidal incisions, likely due to the reduced trauma and improved blood flow associated with this technique (20). This finding is consistent with Mizutani et al. (21), who have observed that less invasive surgical techniques result in better healing outcomes. The choice of incision type is thus crucial for minimizing postoperative complications and should be considered based on patient-specific factors.

In contrast to other studies, this research did not find significant associations between probing depth and complications. Mobadder et al. (22) previously have reported a correlation between deeper probing depths and higher complication rates, but the discrepancy may stem from differences in surgical techniques or patient characteristics. Moreover, while probing depth was significant in the univariate analysis, it lost significance in the multivariate model, which may suggest potential multicollinearity with clinical attachment loss. Future research should further investigate the interaction and relative influence of probing depth and clinical attachment loss on surgical outcomes across different stages of periodontal disease.

The univariate analysis suggested that preoperative antibiotics and analgesics were associated with a reduced risk of postoperative complications, but this effect was not significant in the multivariate analysis, indicating that their protective roles are likely influenced by other clinical or demographic factors. This suggests that antibiotics and analgesics alone may not independently reduce complication rates, and a more comprehensive approach to patient management is necessary. These findings are consistent with previous research showing that while antibiotics and analgesics can reduce complications, their effectiveness depends on factors such as patient age, surgical technique, and overall health (23, 24). Therefore, these interventions should be part of a broader, individualized strategy, and further studies are needed to clarify the conditions in which they provide the most benefit.

Several limitations should be noted. First, this study employed a retrospective design, which limits the ability to establish causal relationships between risk factors and postoperative complications. Second, the study lacked systematic records of patients' perioperative oral hygiene adherence or cleanliness, which may represent important confounding factors influencing wound healing. Third, although smoking was identified as a significant risk factor, no structured smoking cessation interventions were implemented perioperatively. Additionally, the study did not include stratified analyses based on surgical complexity or operator experience, both of which may significantly influence complication rates. Future prospective studies should include larger and more diverse patient populations and consider operator- and procedure-related variables to further validate these findings and assess the long-term outcomes of periodontal flap surgery.

In conclusion, this study identifies age, clinical attachment loss, smoking, and surgical incision type as significant predictors of postoperative complications following periodontal flap surgery. The findings contribute to a deeper understanding of the risk factors involved in periodontal surgery and have important clinical implications. Future research should build on these findings by exploring additional factors that influence healing and developing strategies to optimize postoperative care.

## Conclusion

This study identified age, clinical attachment loss, smoking, and surgical incision type as key risk factors for postoperative complications following periodontal flap surgery. Older age and greater attachment loss were strongly associated with higher complication rates, smoking also increased the risk of complications. Semilunar incisions, typically applied in localized mild cases, were associated with a lower incidence of complications compared to trapezoidal incisions. These findings emphasize the importance of personalized surgical approaches to minimize postoperative complications.

## Data Availability

The original contributions presented in the study are included in the article/Supplementary Material, further inquiries can be directed to the corresponding author.
